# An Adaptive Complex Network Model for Brain Functional Networks

**DOI:** 10.1371/journal.pone.0006863

**Published:** 2009-09-07

**Authors:** Ignacio J. Gomez Portillo, Pablo M. Gleiser

**Affiliations:** Statistical and Interdisciplinary Physics Group, Centro Atómico Bariloche, Bariloche, Río Negro, Argentina; Indiana University, United States of America

## Abstract

Brain functional networks are graph representations of activity in the brain, where the vertices represent anatomical regions and the edges their functional connectivity. These networks present a robust small world topological structure, characterized by highly integrated modules connected sparsely by long range links. Recent studies showed that other topological properties such as the degree distribution and the presence (or absence) of a hierarchical structure are not robust, and show different intriguing behaviors. In order to understand the basic ingredients necessary for the emergence of these complex network structures we present an adaptive complex network model for human brain functional networks. The microscopic units of the model are dynamical nodes that represent active regions of the brain, whose interaction gives rise to complex network structures. The links between the nodes are chosen following an adaptive algorithm that establishes connections between dynamical elements with similar internal states. We show that the model is able to describe topological characteristics of human brain networks obtained from functional magnetic resonance imaging studies. In particular, when the dynamical rules of the model allow for integrated processing over the entire network scale-free non-hierarchical networks with well defined communities emerge. On the other hand, when the dynamical rules restrict the information to a local neighborhood, communities cluster together into larger ones, giving rise to a hierarchical structure, with a truncated power law degree distribution.

## Introduction

Understanding the human brain is one of the greatest challenges in science. A vast diversity of methods have been applied to analyze and study its organization, development and function. Recently, a complex network approach, where the brain is described as a set of vertices and edges, has received much attention [1]–[10]. This interest is due to the fact that the same general principles seem to govern the structural and functional organization of complex networks across a vast diversity of systems, including social, biological and technological networks [11]–[15].

Anatomical studies of the cerebral cortex of mammals such as rat, cat and monkey have shown the presence of highly integrated modules (as one would observe in a regular network) connected sparsely by long range links (giving a short mean distance between nodes across the whole network, as in a random network). This small world structure has been argued to provide an optimal structural substrate which allows for a balance between specialized brain regions and global functional integration [1], [3], [4], [6], [9], [10]. Patterns of functional connectivity have also been observed to present a small-world topology, that seems to be robust across different conditions and measuring techniques [8], [16]–[20]. Recent studies showed that other topological characteristics such as the degree distribution do not seem to be robust, and their functional shape has been a subject of debate. On the one hand, Eguíluz *et al.* [21] used functional magnetic resonance imaging (fMRI) to extract functional networks connecting correlated human brain sites in subjects performing tasks. In these experiments the activity of the brain was measured, in time steps that are spaced 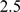 seconds, in a number of “voxels” of dimension 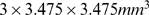. The activity of each voxel 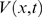 presents a fluctuating oscillatory behavior. By using a correlation measure between any pair of voxels they built a correlation matrix, that was thresholded to construct large-scale brain networks with sizes up to 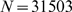 nodes. They found that these are small-world networks with power law degree distributions 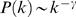, 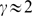. Their results are robust across different subjects, threshold values and task conditions [21]. On the other hand, Achard *et al.* [22] analyzed fMRI time series acquired from healthy subjects in the resting state. Using discrete wavelet transform they obtained frequency-dependent correlation matrices that were thresholded to create undirected graphs with 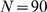 nodes. They found that these networks present a small-world topology in the low-frequency interval 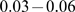 Hz. For the degree distribution they found that the best fit is given by an exponentially truncated power law 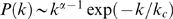 with exponent 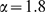 and cutoff degree 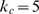, in contrast to the results of Eguíluz *et al.* [21] where no cutoff was observed. Achard *et al.* noted these differences, and suggested that properties of brain functional networks could be conditioned by anatomical resolution of analysis and/or experimental stimulation of the subjects [22]. Finally, we also bring to attention a recent work by Park *et al.* [19], that used diffusion tensor imaging (DTI) and fMRI to analyze functional human brain networks with 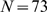 nodes. They obtained a degree distribution that differs from the results of Achard *et al.* [22] and Eguíluz *et al.* [21], presenting a slow decay for small degrees and then a marked crossover to an exponential decay.

The characterization of community structure in functional brain networks also presents different intriguing behaviors. On the one hand, Eguíluz *et al.* [21] obtained networks characterized by a positive correlation between the degrees (indicating the presence of communities) and also with a relative independence of clustering from degree (indicating absence of a hierarchical structure). On the other hand, Ferrarini *et al.* [23] analyzed fMRI images of 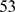 subjects at rest, and using a methodology based on partial correlation analysis [24] extracted brain functional connectivity maps with 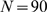 nodes. In contrast to the results of Eguíluz *et al.* [21], they detected overlapping communities, and showed how different regions cluster into larger communities, which then cluster again through a hierarchical organization.

The introduction of theoretical models can shed light into the subject. One possible approach is through realistic models, that include as much detail as possible. On the other extreme, simple models with a minimum number of parameters allow for the determination of the basic ingredients necessary for the emergence of complex structures. In fact, understanding structure function relationships from such a general point of view is an open subject in modern network theory. In particular, much effort is being devoted to the study of synchronization phenomena in populations of elements that are constrained to interact in a complex network topology [25]–[31].

In this work we follow this complex systems approach and present an adaptive complex network model for human brain functional networks. We show that the model is able to describe the topological characteristics of human brain networks obtained from functional magnetic resonance imaging (fMRI) studies [19], [21]–[24], and thus provides a theoretical framework in which to interpret the results. In particular, when the dynamical rules of the model allow for integrated processing over the entire network a scale-free non-hierarchical network with well defined communities emerges. On the other hand, when the dynamical rules restrict the information to a local neighborhood, communities cluster together into larger ones, giving rise to a hierarchical structure, with a truncated power law degree distribution.

In the following section we define the model. Then, in the Results section we present the results of the numerical simulations of the model and compare them with experimental fMRI studies [19], [21]–[24].

## Methods

We modeled a growing adaptive network, where the microscopic units are dynamical nodes that represent different anatomical regions of the brain with their corresponding activity. Starting from a small random network the system grows by the addition of new nodes with a fixed number of connections. These new connections are first established at random, then, an adaptive algorithm allows for rewiring according to the coherence. This algorithm is based on the work of Gong, Van den Berg and van Leeuwen [32]–[34]. They showed that small world networks emerge by adaptively rewiring chaotic units according to their dynamic coherence [33], [34]. A similar form of adaptive evolution was considered in a network of coupled non-linear phase oscillators by Gleiser and Zanette [35], that also found that starting from a random network the system reaches a small-world structure. The fact that an initial random structure is able to spontaneously evolve to a small world network, either when the microscopic units present chaotic or oscillatory dynamics, shows that the algorithm is robust, thus presenting a plausible mechanism for the emergence of complex network structures.

Gong and van Leeuwen showed that scale-free networks emerge when network growth is incorporated to the model with chaotic units [32]. They also found that when the dynamical units are in a 1-period state the degree distribution does not have a scale-free structure, and highlighted the unique importance of chaotic activity for the emergence of scale-free networks. In this work we show that the algorithm is quite robust, and also allows for the emergence of wide degree distributions when the units have a continuous oscillatory dynamics.

The rewiring rules proposed by Gong, Van den Berg and van Leeuwen allow for a global integration of the information of the system, since the state of all the other nodes in the system is available to each new node in order to rewire its links [32]–[34]. This global rewiring dynamics is in some sense optimal, since it allows information on the state of the whole system to be available to each new node. As a consequence, new nodes can always make the best rewiring possible in order to achieve synchronization. We also propose a restricted rewiring dynamics, that only allows for local information to be available to each new node. This allows us to present a theoretical analysis of the effects of a restricted dynamics in the structure-function relationship in the model. In particular, we show that only with this new local rewiring dynamics hierarchical networks emerge.

Let us describe the model in detail. The evolution of the nodes is given by non-linear phase oscillators
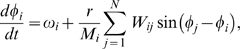
(1)

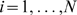
, where 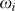 is the natural frequency of oscillator 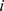 and 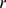 is the coupling strength [28]. The weights 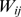 define the adjacency matrix of the interaction network: 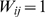 if oscillator 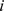 interacts with oscillator 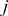, and 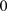 otherwise. The number of neighbours of oscillator 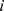 is 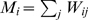. Interactions are symmetric, so that 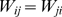 and the network is a non-directed graph.

The model allows for a precise definition of coherence, that reflects the dynamic functional interrelation between spatially separated brain regions, quantified by
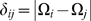
(2)where 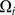 is the average oscillation frequency of oscillator 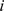 calculated over a time interval of length 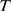,
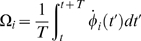
(3)


The algorithm for the evolution of the network is as follows. Begin with a small random network with 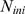 nodes linked by 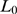 connections per node.

Add a new node 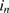 with 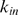 connections to 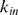 different nodes randomly chosen in the current network.
Calculate the state of the system according to Eq. (1) over a time interval of length 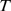. Along this interval calculate 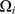 according to Eq. (3).
**a) Global rewiring dynamics**: Calculate the value of 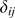 for all 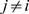. Detect the oscillator 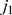 for which 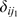 is minimum amongst all the 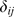. Also detect, amongst the neighbours of 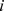, the oscillator 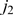 for which 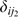 is maximal.
If 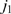 is one of the neighbours of oscillator 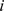, then make no changes to the connections. Otherwise, replace the link between 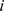 and 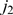 by the link between 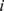 an 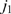.
Go to step 2) and repeat the algorithm for 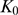 times
Go to step 1)

The Global rewiring dynamics allows for a global integration of the information of the system. We also propose a restricted rewiring dynamics, that only allows for local information to be available:

**b) Local rewiring dynamics**: Calculate the value of 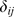 for first and second neighbors of 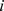. Detect the oscillator 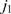 for which 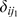 is minimum amongst all the second neighbors of 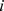. Also detect, amongst the neighbours of 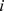, the oscillator 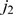 for which 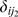 is maximal.

In the following section we show that for global rewiring dynamics scale-free non-hierarchical networks with well defined communities emerges. On the other hand, when the local rewiring dynamics governs the evolution of the system, communities cluster together into larger communities, giving rise to a hierarchical structure, with a truncated power law degree distribution.

## Results and Discussion

The model is quite robust, allowing for a wide range of parameters where the system presents similar characteristics. In order to present a detailed analysis, and a comprehensive comparison with the fMRI studies most parameters will remain fixed and only those ingredients necessary for the formation and evolution of the different network structures will be highlighted. Unless noted, all the results presented in this section correspond to systems with an initial random network with 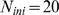 nodes and 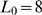 connection per node. The natural frequencies 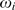 were chosen at random from a Gaussian distribution with zero mean and unitary variance, 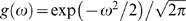. The integration time used to calculate the average oscillation frequency 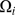 was 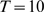, and the coupling strength between the phase oscillators was 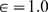. Similar qualitative results were obtained in the numerical simulations for 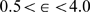.

### Degree Distribution

**Figure 1:**
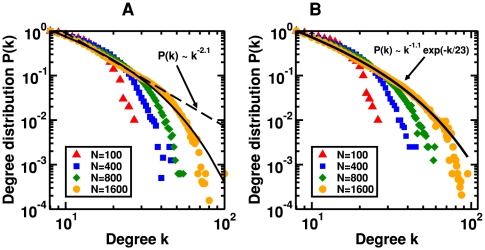
Degree distributions 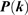 for global (A) and local (B) rewiring dynamics. Degree distribution 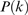 vs. degree 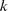 for global (A) and local (B) rewiring dynamics. Four different system sizes 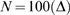, 400(□), 800(◊) and 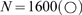 averaged over ten different realizations of the dynamics are shown, when incoming nodes have degree 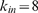 and the coupling strength is 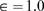. The continuous lines are a guide to the eye and show (A) a power law 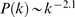 and (B) an exponentially truncated power law 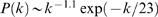.

The algorithm allows for the emergence of wide degree distributions both for global (GRD) and local (LRD) rewiring dynamics. However, the distributions present different functional forms. Figure 1A shows the degree distribution 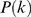 as a function of the degree 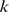 for ten different realizations of GRD and four different system sizes, 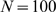, 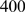, 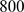 and 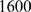 when 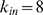. 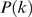 presents a slow decay for small 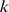 and then a crossover to an exponential decay for large values of 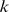. As the system size increases the slower decay extends further and can eventually be fitted by a power law 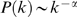 with an exponent 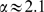 (the straight line in Figure 1A serves as a guide to the eye). This finite size behavior suggests that for large system sizes the crossover will become difficult to observe and the power law behavior will become robust. It is interesting to compare this result with the experimental data of Eguíluz *et al.* [21], where a similar behavior is observed. Using different threshold values they built functional brain networks with large system sizes, ranging from 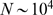 to 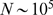, and mean degrees 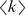 ranging from 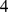 to 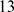 (note that in Figure 1A, 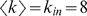). In accordance with the results of the GRD they found power law degree distributions with no cutoff and observed only slight changes in the degree distribution exponents (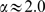) as a function of system size.

For small system sizes the model shows that finite size effects play an important role. Park *et al.* [19] built functional brain networks with only 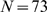 nodes and 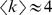, and obtained a degree distribution with a qualitative shape that strongly resembles the results presented in Figure 1A for small system sizes. They observed a slow decay for small 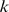 followed by a crossover to an exponential decay (see Figure 1 in [19]). The results of the model suggest that further experimental work including a larger number of functional regions could determine if the crossover observed in [19] is a finite size effect.

Figure 1B shows the behavior of 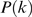 vs. 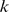 averaged over ten realizations of LRD and four different system sizes, 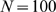, 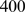, 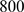 and 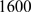 when 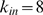. Note that for 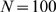 the qualitative shape of 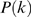 in Figures 1A and B is very similar. This is an expected result, since for small system sizes the LRD and GRD rules will be almost indistinguishable, due to the small mean distance between nodes (since we begin with a random network). However, as 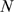 grows the behaviors are different, and the degree distribution for LRD can be fitted by an exponentially truncated power law 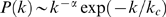 in almost all its 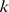 range (the continuous curve in Figure 1B shows the best fit obtained for 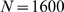). Achard *et al.* [22] observed the same functional form in small networks (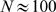 nodes) built from fMRI data of subjects in resting state. They noted that the difference in the functional form of their results (power law with exponential cutoff) and the results of Eguíluz *et al* [21] (power law with no cutoff) could be a consequence of anatomical resolution of analysis and/or experimental stimulation of the subjects [22]. The model highlights the role of anatomical resolution as one of the key ingredients necessary to define the functional shape of the degree distributions.

### Clustering and Hierarchical Structure

**Figure 2:**
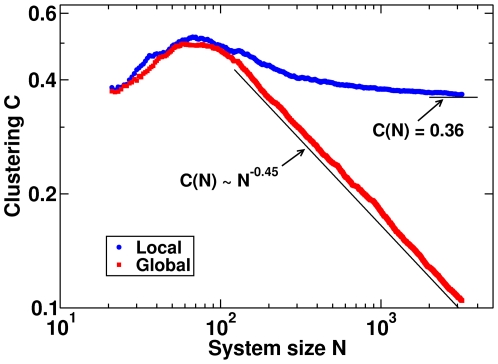
Clustering 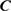 as a function of system size 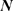 for global and local rewiring dynamics. Clustering 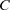 vs. system size 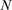 for global (□) and local (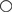) rewiring dynamics, averaged over five samples, when incoming nodes have degree 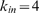 and the coupling strength is 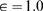. The continuous lines are a guide to the eye and show the power law decay 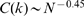 for the global rewiring dynamics and the convergence to the constant value 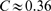 for the local rewiring dynamics.

In order to advance further in the quantitative characterization of the emerging networks we analyzed the clustering coefficient 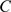, that measures the average number of neighbours of a given node which are in turn mutual neighbors [36]. Ravasz *et al.* [37], [38] have noted that hierarchical networks present a clustering coefficient that is independent of system size. On the other hand, networks that do not present a hierarchical structure (such as the Barabási-Albert model for scale-free networks [38]) present a decaying behavior of 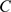 with 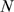. Since the model allows for such a finite size study, we present in Figure 2 the behavior of 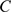 as a function of 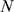 for GRD (□) and LRD (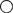), when 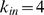. The curves correspond to averages over five different networks. Note that 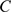 presents a non-monotonic behavior, and, as expected, the behavior for small 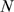 is similar for both dynamics. As 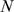 grows the curves depart, and for GRD the clustering coefficient decays following approximately a power law 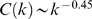 (the straight line in figure 2 is a guide to the eye), while for LRD the clustering coefficient seems to converge to an asymptotic constant value 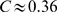. This striking difference between the two dynamics shows that the characteristics of a hierarchical network are present for LRD, while they are clearly absent for GRD.

**Figure 3:**
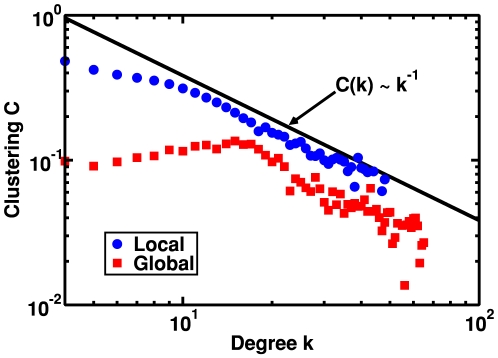
Clustering 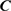 as a function of degree 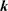 for global and local rewiring dynamics. Clustering 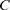 vs. degree 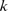 for global (□) and local (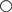) rewiring dynamics, averaged over five samples, when incoming nodes have degree 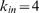, the coupling strength is 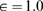 and 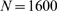. The straight line is a guide to the eye and shows the power law behavior of a hierarchical network, 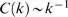.

In Figure 3 we compare the behavior of 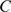 as a function of 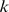 for both rewiring dynamics when 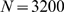 and 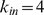. The curves were averaged over five different networks. For GRD (□) the behavior of 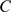 is almost constant for degrees up to 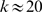, and then presents a slow decay for larger values of 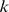. A qualitatively similar behavior was obtained by Eguíluz *et al.* (see Figure 5 in [21]), and was interpreted as an absence of hierarchical organization, where a power law decay 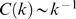 was expected [38] (this behavior is presented as a guide to the eye in Figure 3). For LRD (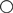) the behavior of 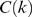 is qualitatively similar, however we should stress that the clustering coefficient presents larger values in the whole 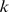 range, and deviations between the two dynamics are noticeable for nodes with small degree. In the following section we will comment on the origin of these differences.

Ferrarini *et al.* [23] obtained functional brain networks that present hierarchical structure. They highlight that their method allows them to detect overlapping communities, showing how different regions cluster into larger ones, which then cluster again through a hierarchical organization [23]. In the following section we will analyze the synchronization properties of the system. In particular, we will establish a relation between the formation and organization of synchronized clusters and the underlying network structure. This will allow us to understand the mechanisms that lead to the formation (absence) of a hierarchical structure when LRD (GRD) is considered.

### Synchronization

**Figure 4:**
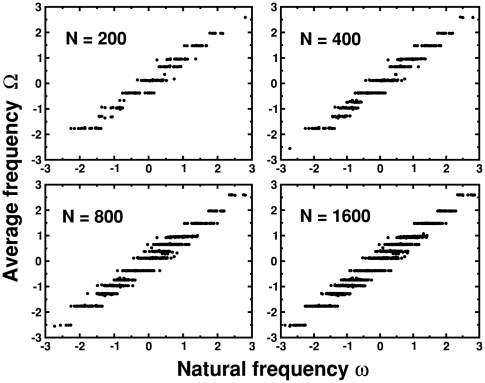
Evolution of synchronized clusters for global rewiring dynamics. Average frequency 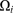 vs. natural frequency 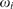 for global rewiring dynamics for four different system sizes 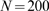, 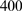, 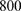 and 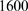 of a single sample. Horizontal arrays of dots indicate synchronized clusters.

In order to establish the interplay between the collective dynamics of the oscillators and the underlying network structure we analyzed first the formation and evolution of synchronized clusters. Figure 4 shows the behavior of average frequencies 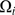 as a function of natural frequencies 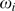 for GRD for four different system sizes, 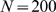, 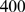, 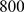 and 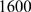. Already for 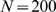 the presence of a number of horizontal arrays of dots can be clearly seen. They indicate that oscillators with different natural frequencies have attained the same average frequency, showing that they form a synchronized cluster. Note that for increasing 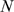 the number of clusters remains almost constant, and thus only their size grows. This behavior reflects the global character of the dynamics: once the synchronized clusters are formed, the connections of the new nodes can rewire to any of the synchronized clusters, and thus choose the one with average frequency closer to its natural frequency.

The formation and evolution of groups of synchronized oscillators reveal a non-trivial underlying network structure. In fact, it allows for an explanation of the non-monotonic behavior of 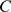 vs 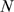 observed in Figure 2. Initially the system is a small random network with a small clustering. As the system size grows synchronized clusters formed by oscillators that are connected between themselves emerge, and as a consequence the clustering grows. Eventually, since the number of synchronized clusters remains constant, only their size grows, as a consequence the connections between the nodes in a given cluster become sparse, and the clustering decays.

**Figure 5:**
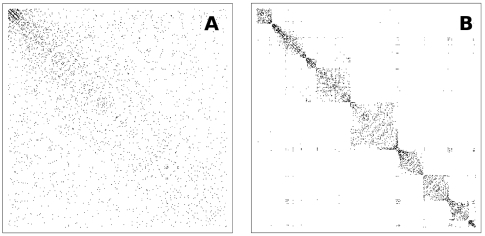
Adjacency matrix for global rewiring dynamics. Adjacency matrix for global rewiring dynamics when the nodes are ordered according to: (A) the time in which they were incorporated into the system, (B) the value of their average frequency 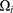.

We also analyzed the evolution of the network structure. In Figure 5A we present the adjacency matrix 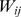 for a system with 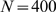 using GRD. Each dot in the matrix corresponds to a connection (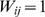) between nodes 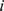 and 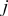. The axes have been ordered according to the time in which the nodes entered the system. Note that for small values of 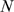 (short times) the matrix is dense, becoming sparser as 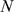 grows. The adjacency matrix also allowed us to establish the interplay between the synchronized groups and the topological structure of the network. In Figure 5B the same matrix has been reordered according to the average frequency of the nodes in increasing order. Note the presence of well defined communities around the diagonal, that have a direct relation to the synchronized clusters observed in Figure 4. Also note the presence of few connections far from the diagonal line, outside the modules. These correspond to oscillators that have long-range connections linking different synchronized modules and reflect the small-world character of the network. Again the results obtained with GRD present a strong resemblance with the experimental work of Eguíluz *et al.* [21], that observed an assortative mixing in their functional brain networks, a clear indication of the presence of communities [39].

**Figure 6:**
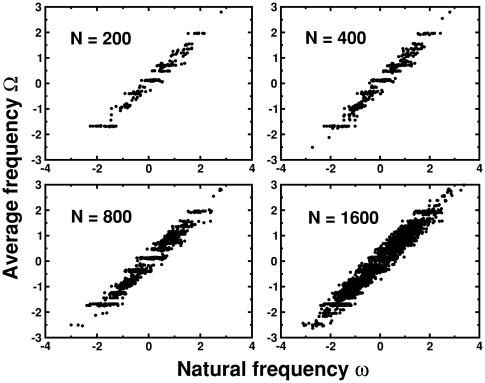
Evolution of synchronized clusters for local rewiring dynamics. Average frequency 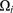 vs. natural frequency 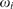 for local rewiring dynamics for four different system sizes 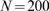, 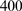, 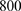 and 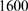 of a single sample. Horizontal arrays of dots indicate synchronized clusters.

For LRD the formation and evolution of synchronized clusters presented a different behavior. Figure 6 shows the behavior of 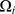 as a function of 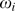 for LRD for four different system sizes, 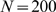, 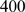, 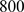 and 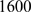. As expected, for small system sizes the local and global rewiring dynamics present similar results, and a number of synchronized clusters can already be seen for 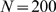. Note however, that the clusters are not clearly separated as in figure 4. As the system size grows new horizontal arrays of dots appear between the clusters, indicating the formation of new groups of synchronized oscillators. Eventually, it is very difficult to separate the different horizontal arrays that are distributed along the whole frequency range.

**Figure 7:**
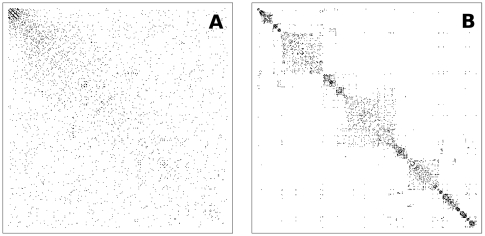
Adjacency matrix for local rewiring dynamics. Adjacency matrix for local rewiring dynamics when the nodes are ordered according to: (A) the time in which they were incorporated into the system, (B) the value of their average frequency 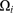.

In order to establish the interplay between this new synchronization behavior and the underlying network structure, we also analyzed the evolution of the network topology through the adjacency matrix. In Figure 7A we show the adjacency matrix 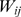 for a system grown with LRD with 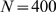 nodes, where the axes label the nodes according to the time in which they entered the system. Note that, as with GRD (Figure 7A) for small values of 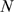 the matrix appears dense, becoming sparser for larger 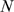. In Figure 7B, the matrix was reordered according to the average frequency 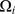 in increasing order. In this case, the identification of synchronized clusters with the topological structure of the network revealed the presence of overlapping communities, that cluster into larger ones through a hierarchical organization, presenting a strong resemblance to the results of Ferrarini *et al.* [23] and also to the adjacency matrices obtained by Achard *et al.* (see Figure 1 in [22]).

In this section we have analyzed the synchronization properties of the model, and also its relation with the underlying network structure. We showed that the global rewiring dynamics allows for the formation of clusters of synchronization that have a direct relation with the formation and evolution of network communities. This dynamics is an optimal rewiring dynamics, in the sense that it allows each new node that enters the system to make the best rewiring possible given the state of the system. That is, to rewire all its connections to the synchronized cluster that has an average frequency closer to its natural frequency. This mechanisms limits the formation of new synchronized clusters, since once they are formed they only grow in size. The same behavior is reflected in the underlying network structure, where a limited number of communities are formed that become sparser as the system size grows.

On the other hand, when the rewiring rules are restricted to a local neighborhood, each new node cannot always choose the best rewiring to synchronize with a given cluster, and thus may end up with an average frequency different from the synchronized clusters that were present. This allows for the formation of new clusters with new average frequencies. As the system grows new synchronized clusters emerge and grow at different average frequencies. The underlying network structure reflects this behavior by the emergence of communities that cluster together into larger ones, giving rise to a hierarchical structure.

Summarizing, in order to understand the basic ingredients necessary for the emergence of the complex network structures observed in human brain functional networks, we presented an adaptive complex network model. The microscopic units of the model are dynamical nodes, and the links between the nodes are chosen following an adaptive algorithm that allows for rewiring between dynamical elements with similar internal states. We have shown that the model is able to describe topological characteristics of human brain networks obtained from functional magnetic resonance imaging studies. In particular, when the dynamical rules of the model allow for integrated processing over the entire network scale-free non-hierarchical networks with well defined communities emerge, resembling the experimental results of Eguíluz *et al.* [21]. On the other hand, when the dynamical rules restrict the information to a local neighborhood, communities cluster together into larger ones, giving rise to a hierarchical structure, with a truncated power law degree distribution, resembling the experimental results of Achard *et al.* [22] and Ferrarini *et al.* [23].
